# Footwear and Foam Surface Alter Gait Initiation of Typical Subjects

**DOI:** 10.1371/journal.pone.0135821

**Published:** 2015-08-13

**Authors:** Marcus Fraga Vieira, Isabel de Camargo Neves Sacco, Fernanda Grazielle da Silva Azevedo Nora, Dieter Rosenbaum, Paula Hentschel Lobo da Costa

**Affiliations:** 1 Bioengineering and Biomechanics Laboratory, Universidade Federal de Goiás, Goiânia, Goiás, Brazil; 2 Physical Therapy, Speech, and Occupational Therapy Department, School of Medicine, Universidade de São Paulo, São Paulo, Brazil; 3 Institute for Experimental Musculoskeletal Medicine, Movement Analysis Lab, University Hospital, Münster, Germany; 4 Physical Education Department, Universidade Federal de São Carlos, São Carlos, Brazil; The University of Queensland, AUSTRALIA

## Abstract

Gait initiation is the task commonly used to investigate the anticipatory postural adjustments necessary to begin a new gait cycle from the standing position. In this study, we analyzed whether and how foot-floor interface characteristics influence the gait initiation process. For this purpose, 25 undergraduate students were evaluated while performing a gait initiation task in three experimental conditions: barefoot on a hard surface (barefoot condition), barefoot on a soft surface (foam condition), and shod on a hard surface (shod condition). Two force plates were used to acquire ground reaction forces and moments for each foot separately. A statistical parametric mapping (SPM) analysis was performed in COP time series. We compared the anterior-posterior (AP) and medial-lateral (ML) resultant center of pressure (COP) paths and average velocities, the force peaks under the right and left foot, and the COP integral x force impulse for three different phases: the anticipatory postural adjustment (APA) phase (Phase 1), the swing-foot unloading phase (Phase 2), and the support-foot unloading phase (Phase 3). In Phase 1, significantly smaller ML COP paths and velocities were found for the shod condition compared to the barefoot and foam conditions. Significantly smaller ML COP paths were also found in Phase 2 for the shod condition compared to the barefoot and foam conditions. In Phase 3, increased AP COP velocities were found for the shod condition compared to the barefoot and foam conditions. SPM analysis revealed significant differences for vector COP time series in the shod condition compared to the barefoot and foam conditions. The foam condition limited the impulse-generating capacity of COP shift and produced smaller ML force peaks, resulting in limitations to body-weight transfer from the swing to the support foot. The results suggest that footwear and a soft surface affect COP and impose certain features of gait initiation, especially in the ML direction of Phase 1.

## Introduction

To start a new walking cycle, anticipatory postural adjustments (APAs) are necessary for stabilizing the postural perturbation induced by the forthcoming voluntary movement [1, 2]. Therefore, gait initiation is the functional task used to understand the anticipatory postural adjustments needed to execute the transition from standing posture to cyclic walking [3–5]. The role of gait initiation is to generate the force and impulse necessary to move the center of pressure (COP) toward the swing foot and then toward the support foot in order to enable a safe step [6] and can be divided in three phases [7]:

- the APA phase or swing-foot loading phase (Phase 1), consists of a backward and lateral displacement of the COP toward the swing foot before any observable movement of the feet. This anticipatory adjustment protects the body from balance disturbances and at the same time generates the forward impulse for the forthcoming progression [8],
- the swing-foot unloading phase (Phase 2), consists of the COP movement toward the support foot, starting with the swing-foot heel-off and ending with the ipsilateral toe-off. In this phase, the COP path corresponds to the vertical projection of the center of mass (COM) and
- the support-foot unloading phase (Phase 3), consists of the COP movement forward under the support foot. This phase corresponds to the initial single support phase followed by the double support phase of the first step, ending with toe-off of the support foot.

Gait initiation is an interesting process to be studied from the fundamental point of view, because it involves a precise coordination between posture and movement in order to feedforward control the postural adjustments needed to start a new gait cycle, as well as from the clinical one, because imbalances during gait initiation have been shown to discriminate between stable and unstable patients [9]. Especially regarding healthy older adults, a fear-of-falling group produced longer gait initiation phases than a non-fear-of-falling group, even though both groups were not different when tested with functional balance measurements [10].

The use of footwear changes the sensory input to the motor control system, which in turn modifies the motor responses and adjustments [11,12]. Footwear may also reduce the range of motion of the foot and ankle and thereby partially block the foot rollover process, altering the rocker action of the foot and ankle [13] and causing a loss of the eccentric ankle control occurring from the heel-strike to the flatfoot phase [14]. The natural range of motion of the forefoot relative to the rearfoot is decreased during shod locomotion [15], especially the eversion/inversion amplitude [16]. Furthermore, when comparing walking and running in three different conditions (wearing hard shoes, wearing soft shoes and barefoot) it was found significant differences in the coordinative ankle strategy between shoe conditions and barefoot [17].

There is evidence in the literature [18–21] showing the benefit of the better sensory perception provided by barefoot locomotion. The greater amount of sensory feedback coming from the foot during ground contact triggers neuromuscular reflexes that help to minimize proximal joint loads [21]. During barefoot walking, the longitudinal plantar arch appears to be higher, which potentially results in an enhanced load accommodation, probably due to a motor strategy of changing the plantar architecture for better shock absorption [22]. Sensory information from the plantar surface during ground contact is essential to these foot adaptations and may be weakened when using footwear [22] or when walking over a soft surface.

The instant prior to the first contact of the swing foot is recognized as the most challenging event for stability control during gait initiation [9]. Accordingly, Chastan et al. [23] showed that depending on the hardness of the surface and the presence of visual feedback during walking, the active breaking of the downward movement of the COM changes significantly in gait initiation, particularly during the transition to double support.

The gait initiation process is completed when the body reaches a constant walking speed, roughly between the beginning of the third and the end of the eighth step [24]. Footwear type has been shown to influence the number of steps necessary to reach a steady-state gait: using habitual shoes with foot orthoses may help the body reach steady-state walking with fewer steps than do barefoot walking and wearing habitual shoes without orthoses [25]. Medial-lateral COM range of motion during gait initiation is reduced when foot orthoses are used, compared to barefoot walking and the use of habitual shoes, and this advantage has been related to a higher level of stimulation of mechanoreceptor activity with the foot orthoses and improved dynamic postural stability [25]

Some experiments have also demonstrated that the manipulation of different sensory modalities has an important influence on postural adjustments during gait initiation. Cutaneous stimulation (low-frequency vibration) of the ankle muscles changes the anticipatory postural adjustment phase in healthy young subjects [26]. Reduction in the afferent somatosensory information under the foot by means of immersion in iced water results in a reduced medial-lateral COP excursion during the anticipatory phase, and this loss of sensitivity is not compensated by visual input [27], indicating that plantar cutaneous afferents contribute to the control of gait initiation.

Therefore, somatosensory information related to both the textural characteristics of the support surface and the use of footwear may play a role throughout the process of gait initiation, because the foot/ankle mechanical features can be modified and different sensory information can be used as feedback to the central nervous system.

Although it has been studied extensively in Parkinson's patients [3,28–33], elderly people [10,34–37], healthy and pathological children [8,38–44], and amputees [45–49] and under several environmental constraints [26,27,50–53], evidence about whether foot-floor interfaces influence gait initiation in typical subjects is still lacking in the literature. Since the mechanism to propel the body while maintaining balance is regulated by stereotyped activities of ankle muscles [54] and important contributions of plantar cutaneous afferents [27], it is likely that the gait initiation process is affect by different foot support characteristics, because they would affect both muscle activity and somatosensory information.

Thus, the aim of the present study is to investigate the influence of footwear and of a soft surface on gait initiation. We analyzed COP and ground reaction forces time series during each gait initiation phase in three conditions—barefoot on a hard surface, barefoot on a soft surface, and using a habitual shoe—under the hypothesis that compared to the barefoot condition, footwear and a soft surface will limit anticipatory postural adjustment amplitudes, changing COP behavior. For this purpose, we used classical variables, i.e. COP path and mean COP velocity, discrete variables, such as force peaks, to identify differences between conditions and for a proper comparison with previous results, and force impulse x COP integral to analyze the role of COP displacement in body weight transfer between swing foot and support foot [34]. In addition, to capture features of the entire COP time series instead of a few discrete variables, we conduct a vector analysis using statistical parametric mapping (SPM) methods [55].

## Methods

### Subjects and Ethics Statement

Twenty-five undergraduate students of both sexes (24.4 ± 6.2 years old, 69.4 ± 12.7 kg, 1.7 ± 0.1 m) were enrolled in the study. All students were healthy, without any musculoskeletal injury or pain at the time of data collection.

The students underwent medical examinations and were fully informed about the procedures and risks involved. They were allowed to leave the study at will and to opt out of any of our tests. They voluntarily signed an informed consent form, specifically approved for this study, prior to participation. The study was approved by the institutional Ethics Committee for Human Research of the Universidade Federal de Goiás, Brazil (Approval Number 271/2011).

### Experimental Procedure

Ground reaction forces and moments were collected using two synchronized AMTI force platforms (OR6-7 model) positioned at the beginning of an 8-m walkway.

From a standing position, with one foot on each platform and upper limbs alongside the trunk, the subjects executed four trials in the following experimental conditions: barefoot on a hard surface (force platform), barefoot on a soft surface (foam—dimension: 100x50x3 cm, density: 20 kg/m^3^, elastic modulus: 9000 N/m^2^), and habitual shod. The foam covered only the force platforms. The experimental conditions were randomly assigned to each subject.

After a verbal command, the subject started to walk with their preferred foot (right foot for all subjects) and a self-selected velocity to the end of the walkway, performing two complete gait strides. We assigned force platform 1 to the swing foot (right foot) and force platform 2 to the support foot (left foot).

The data acquisition started two seconds prior to the verbal command, and the data were collected at 100 Hz. A resting period of 30 s between each condition was allowed.

A print of the support base and foot was taken to calculate the support-base width and the foot length and width and to standardize the position of the subject on the force platform.

### Mathematical and Statistical Analysis

The data were filtered using a fourth-order zero-lag low-pass Butterworth filter, with a cutoff frequency of 6.0 Hz. In the COP calculations, the average thickness of the foam when the subject was standing on it (0.01 m) was added to the vertical coordinate of the force platform’s actual origin (provided by the manufacturer), as presented in Eqs 1 and 2:

$$COP_{AP}=\frac{M_{x}-(c-\tau )F_{y}}{F_{z}}+b$$ (1)

$$COP_{ML}=\frac{-M_{y}-(c-\tau )F_{x}}{F_{z}}+a$$ (2)

where COP_AP_ and COP_ML_ are the anterior-posterior (AP) and medial-lateral (ML) components of the COP time series, respectively; F_x_, F_y_, and F_z_ are the medial-lateral, anterior-posterior, and vertical ground reaction force components, respectively; a, b, and c are the x, y, and z coordinates of the force platform’s actual origin; and τ is the thickness of the foam placed on the force platform.

The resultant COP between the two force platforms was calculated as follows:

$$R_{esul\text{tan}t}COP_{AP}^{}=COP_{AP1}^{}\frac{F_{z1}^{}}{F_{z1}^{}+F_{z2}^{}}+COP_{AP2}^{}\frac{F_{z2}^{}}{F_{z1}^{}+F_{z2}}$$ (3)

and

$$R_{esul\text{tan}t}COP_{ML}^{}=COP_{ML1}^{}\frac{F_{z1}^{}}{F_{z1}^{}+F_{z2}^{}}+COP_{ML2}^{}\frac{F_{z2}^{}}{F_{z1}^{}+F_{z2}}$$ (4)

where indexes 1 and 2 refer to force platform 1 and force platform 2, respectively.

**Fig 1:**
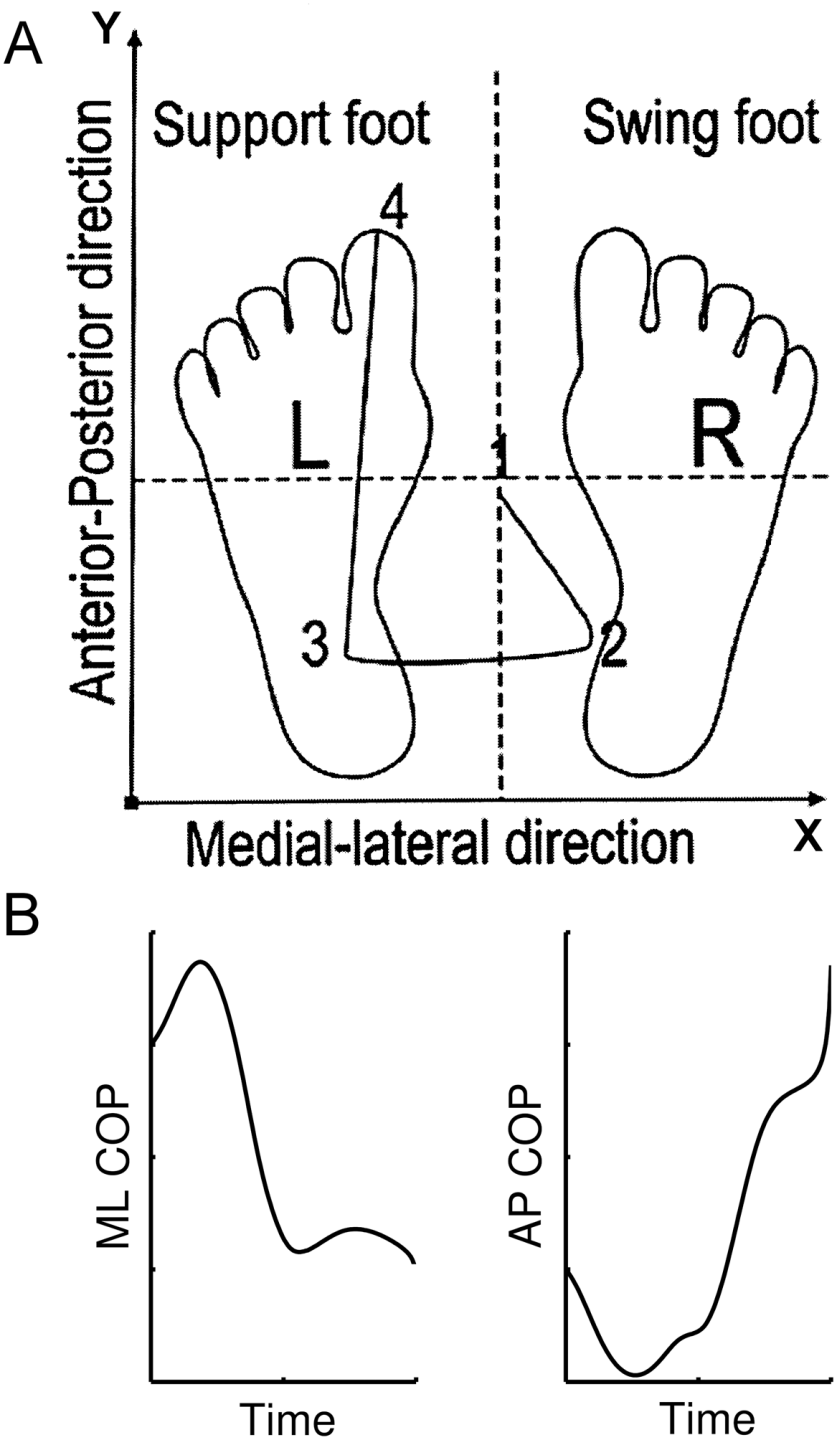
Typical COP path during gait initiation. Above: Resultant COP time series. R: right foot, L: left foot. 1–2: Phase 1 (APA phase); 2–3: Phase 2 (swing-foot unloading phase); 3–4: Phase 3 (support-foot unloading phase). Below—Left: ML COP path, Right: C: AP COP path.

The resultant COP time series was divided into three phases, as follows [7] (Fig 1):

- Phase 1, defined as the interval between the instant when the vertical force of force platform 1 exceeds its mean value plus two standard deviations in the 0.5 seconds prior to the verbal command [56, 57] and the instant when the COP reaches the most lateral position toward the swing foot.
- Phase 2, defined as the interval between the end of Phase 1 and the instant when the swing-foot loses contact with force platform 1 (the vertical reaction force of force platform 1 is lower than a threshold). The threshold, adopted for all subjects, was calculated as 2% of the average subjects’ body weight, because the COP is sensitive to errors in forces and to moments when F_z_ assumes very small values, that is, at the initial and final foot contact during a step.
- Phase 3, defined as the interval between the end of Phase 2 and the instant when the support foot loses contact with force platform 2 (the vertical reaction force of force platform 2 is lower than a threshold, as defined above).

A custom-written Matlab code was used to compute the instantaneous resultant COP, its phases, and the resultant COP descriptor variables in the AP and ML directions: (1) COP path: calculated as the cumulative sum of the displacements at each sampling period between the beginning and the end of each phase (PathML and PathAP); (2) COP mean velocity: calculated as the ratio between COP path and the duration of the corresponding phase (VelML and VelAP).

In order to estimate the relative contribution to weight transfer between swing and support foot [38], the force peaks normalized by body weight were also compared separately for the swing and support foot in Phases 1, 2, and 3. For this purpose, the highest vertical force peak between the two platforms was taken, and then the others force peak values (AP and ML components) were calculated considering the instant at which this highest vertical force peak was observed in each phase.

Furthermore, the time integral of the resultant COP time series in both AP and ML directions in Phases 1 and 2 was compared to the amount of impulse computed by integrating the corresponding ground reaction force component over the same time interval [34].

The COP time series were normalized such that at the beginning of the task, the AP and ML COP components were equal to zero. The ML COP component was normalized by the support-base width or foot width (resultant COP and COP on each force platform, respectively) and the AP COP component by the foot length.

In addition, a vector analysis was conducted in the resultant COP time series using the SPM method, as described by Pataky et al. [55]. This statistical approach captures features of the entire time series instead of a few discrete variables and may improve the effectiveness of the gait initiation analysis. Discrete variables fail to capture sufficient portions of the data and covariance among vector components [55]. SPM analysis uses random field theory to identify field regions which co-vary with the experimental protocol [58].

Each component of the resultant COP time series was interpolated with cubic splines to contain 100 points. The average of the trials was used in the analysis. Next, the components were organized in a matrix with 25 rows, one for each subject, and 200 columns (100 from each component). One matrix was constructed for each condition.

Each column of the (25 x 200) COP matrices was regarded as a single-vector field Cop(t) = {Cop_x_(t) Cop_y_(t)}, where t indicates time. Within-subject mean Cop(t) was calculated for each i^th^ subject at each time instant and among the three conditions, taken two by two:

$$\Delta Cop{\left(t\right)}_{i}={\left(Cop{\left(t\right)}_{condition1}\right)}_{i}-{\left(Cop{\left(t\right)}_{condition2}\right)}_{i}$$ (5)

where conditions are barefoot, foam and shod.

The paired Hotellings *T* ^*2*^ test statistic trajectory was calculated as:

$$T^{2}(t)=n(\Delta \bar{C}op(t))W{\left(t\right)}^{-1}(\Delta \bar{C}op{\left(t\right)}^{T})$$ (6)

where n is the number of subjects (number of rows), $\Delta \bar{C}op(t)$ is the cross-subject mean, and W(t) is the 2 x 2 covariance matrix of Δ*Cop*_*x*_ and Δ*Cop*_*y*_ at time t.

For vector analysis, statistical inference was conducted by calculating the *T* ^*2*^ threshold taking α = 0.05. Conditions effects were assessed using paired *t* tests on COPx, COPy with a Sidák correction threshold taking α = 0.05 producing p calculated as follows:

pcritical=1−(1−α)1N (7)

where N = 2 is the number of scalars for COP (x and y). Eq 7 produces p = 0.0253 for N = 2.

A custom-written Matlab code, based on Pataky [59], was used to conduct SPM analysis.

For the resultant COP variables and force peaks of the swing and support foot, due to the Gaussian data distribution (Shapiro-Wilk test, p > 0.05), repeated measures analysis of variance (ANOVA) was applied to assess the effects of different conditions, followed by Newman-Keuls post hoc tests and the Bonferroni correction, respectively. For COP integral and force impulse, a linear regression was calculated by least squares fitting, and Spearman’s rank correlations were used to explore the relationship between these measures. The statistical analysis was performed with SPSS software (version 17, SPSS Inc., Chicago, IL), with a significance level set at p < 0.05.

## Results

**Fig 2:**
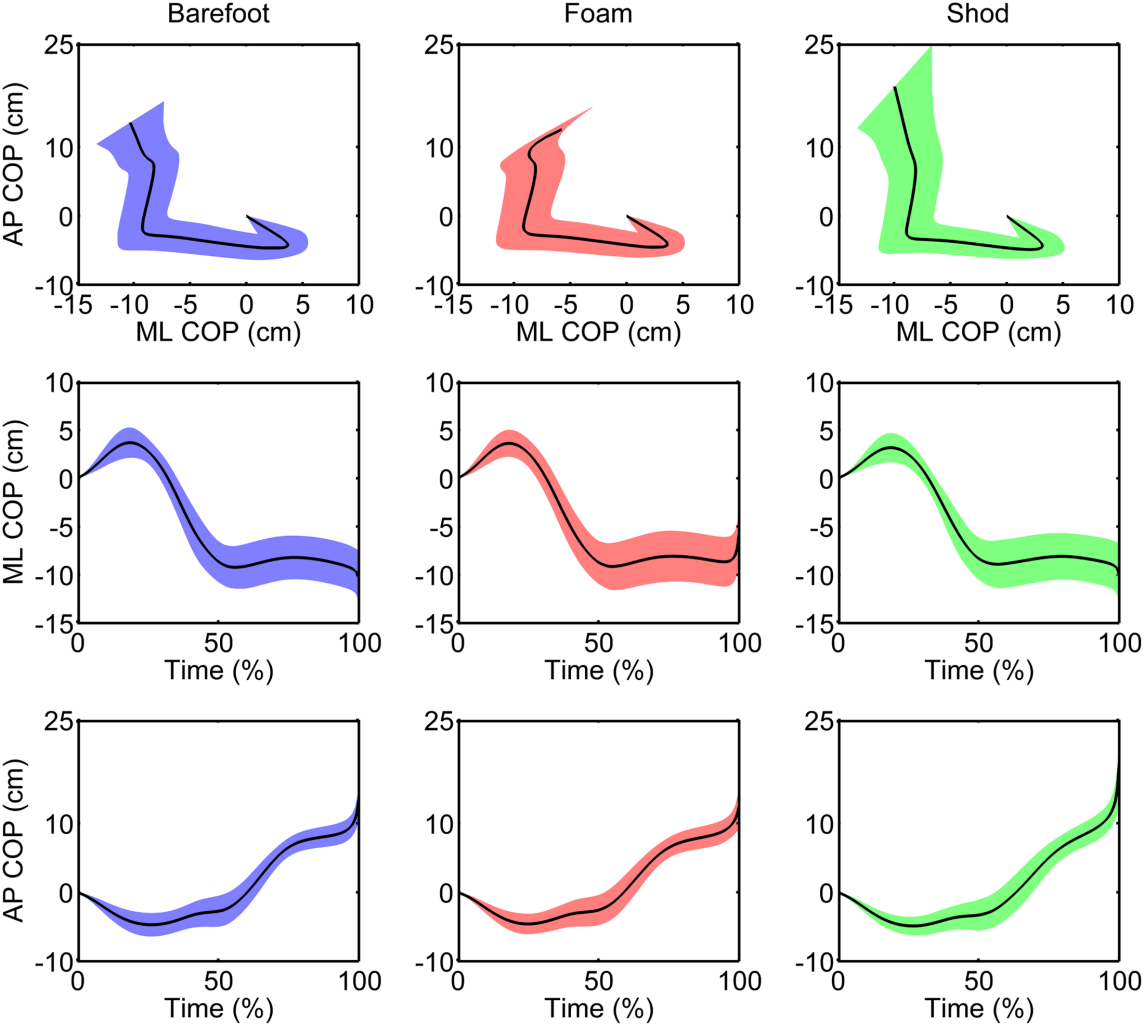
Mean COP path time series in Barefoot, Foam and Shod conditions. Top graphs: Resultant COP path, Middle graphs: ML COP path, Bottom graphs: AP COP path.

The mean COP time series are presented in Fig 2. For resultant COP variables in Phase 1, barefoot and foam conditions produced significantly increased COP paths (p = 0.002 and p = 0.007, respectively) and COP velocities (p = 0.007 and p = 0.015, respectively) in the ML direction compared to the shod condition (Table 1). In Phase 2, the only significant difference was found for the ML COP path, where the barefoot and foam conditions produced increased paths compared to the shod condition (p = 0.016 and p = 0.044, respectively). The barefoot and foam conditions were not significantly different in this phase for any variable tested.

**Table 1:** Resultant COP Descriptive Variables in All Three Phases of Gait Initiation.

		*Barefoot*	*Foam*	*Shod*
***Phase 1***	PathML	0.15 ± 0.06^a^	0.16 ± 0.05^b^	0.13 ± 0.06^a^ ^,^ ^b^
	PathAP	-0.17 ± 0.060	-0.18 ± 0.10	-0.17 ± 0.05
	VelML	1.01 ± 0.55^c^	1.02 ± 0.53^d^	0.85 ± 0.47^c^ ^,^ ^d^
	VelAP	-1.12 ± 0.63	-1.13 ± 0.63	-1.14 ± 0.50
***Phase 2***	PathML	0.52 ± 0.14^e^	0.54 ± 0.10^f^	0.48 ± 0.15^e^ ^,^ ^f^
	PathAP	0.15 ± 0.06	0.17 ± 0.06	0.15 ± 0.07
	VelML	2.14 ± 1.00	2.15 ± 0.91	1.96 ± 0.91
	VelAP	0.56 ± 0.20	0.73 ± 0.45	0.55 ± 0.24
***Phase 3***	PathML	0.18 ± 0.08	0.22 ± 0.07	0.16 ± 0.06
	PathAP	0.65 ± 0.17^g^	0.62 ± 0.17^h^	0.88 ± 0.26^g^ ^,^ ^h^
	VelML	0.52 ± 0.34	0.57 ± 0.28	0.44 ± 0.28
	VelAP	1.76 ± 0.75^i^ ^,^ ^j^	1.61 ± 0.74^i^ ^,^ ^k^	2.45 ± 1.36^j^ ^,^ ^k^

Values expressed as mean ± standard deviation. Repeated measures ANOVA—pairwise comparisons:

^a^ p = 0.002,

^b^ p = 0.007,

^c^ p = 0.007,

^d^ p = 0.015,

^e^ p = 0.016,

^f^ p = 0.044,

^g^ p < 0.001,

^h^ p < 0.001,

^i^ p = 0.024,

^j^ p < 0.001,

^k^ p < 0.001.

In Phase 3, significantly increased COP paths in the AP direction were found for the shod condition compared to the barefoot and foam conditions (p < 0.001 and p < 0.001, respectively), and significantly increased mean velocity was also found (p < 0.001 and p = 0.001, respectively). In some respects, these COP path results were expected, because footwear makes the foot segment larger in the shod condition than in the other conditions and the data were normalized by foot length. Nonetheless, the significantly higher AP mean velocity for the shod condition indicates that this phase is shorter when footwear is used. Furthermore, the velocity in the AP direction was significantly lower in the foam condition compared to the barefoot condition (p = 0.024).

**Fig 3:**
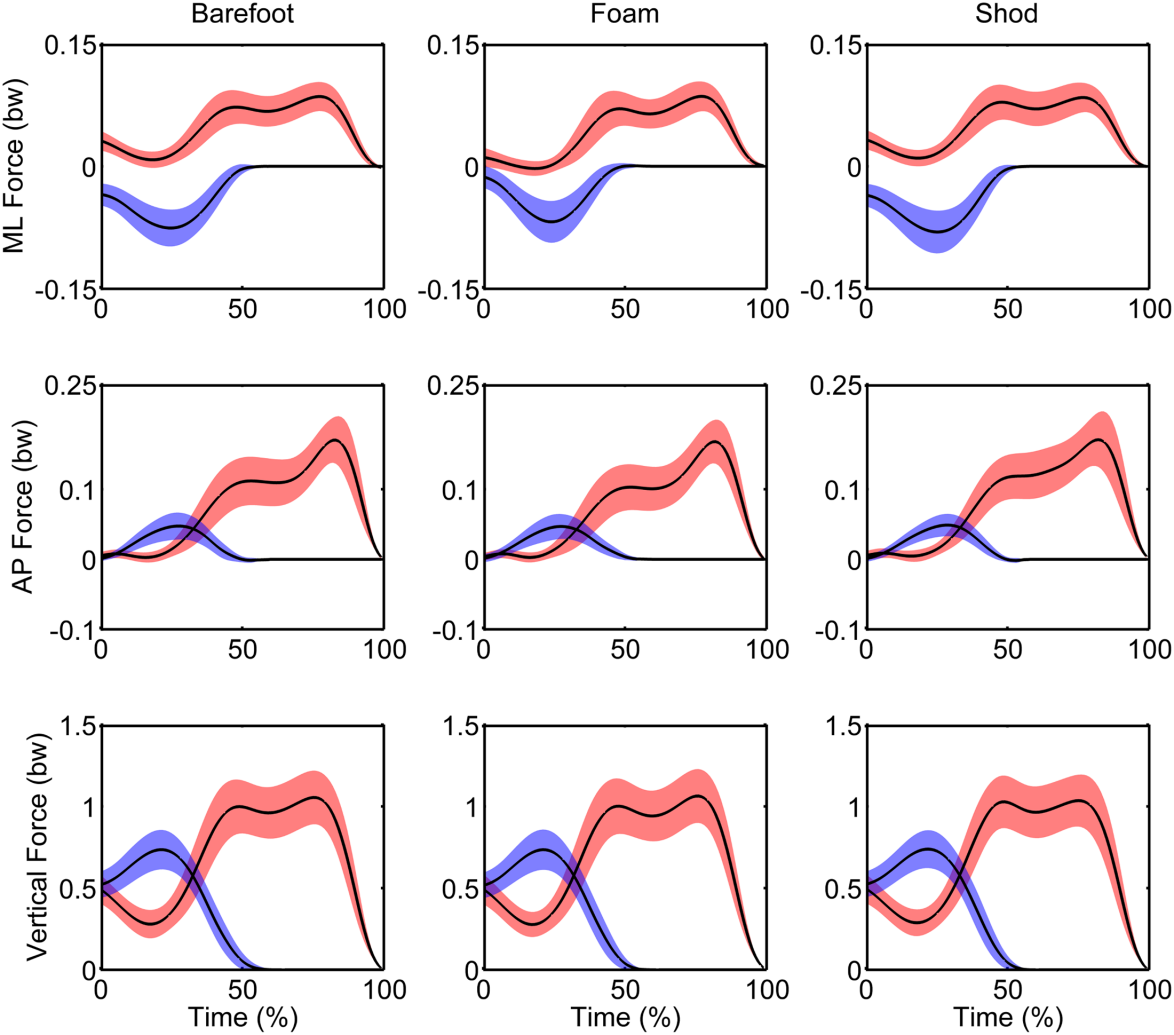
Mean force time series in the Barefoot, Foam and Shod conditions. Blue: swing foot, Red: support foot. bw: body weight.

The relative contribution of each foot to the swing-foot loading and unloading processes is presented in Tables 2–4 and the force time series are presented in Fig 3. In Phase 1 (Table 2), the swing- and support-foot force peaks in the ML direction were significantly smaller in the foam condition than in the barefoot (p = 0.002, p < 0.001, respectively) and shod conditions (p < 0.001, p < 0.001, respectively). In all directions, force peaks were larger under the swing foot than under the support foot in Phase 1, indicating strong swing-foot loading.

**Table 2:** Force Peaks during Phase 1.

	*Barefoot*		*Foam*		*Shod*	
	Swing	Support	Swing	Support	Swing	Support
***ML force***	-0.068 ± 0.015^a^	0.007 ± 0.009^c^ ^,^ ^d^	-0.058 ± 0.016^a^ ^,^ ^b^	-0.003 ± 0.009^c^ ^,^ ^e^	-0.069 ± 0.013^b^	0.009 ± 0.009^d^ ^,^ ^e^
***AP force***	0.037 ± 0.014	0.002 ± 0.007	0.035 ± 0.013	0.002 ± 0.007	0.036 ± 0.013	0.004 ± 0.008
***Vertical force***	0.71 ± 0.05	0.26 ± 0.07	0.71 ± 0.05	0.26 ± 0.06	0.70 ± 0.04	0.27 ± 0.06

Values expressed as mean ± standard deviation. Repeated measures ANOVA pairwise comparisons:

^a^ p = 0.002,

^b^ p < 0.001,

^c^ p < 0.001,

^d^ p = 0.007,

^e^ p < 0.001.

**Table 3:** Force Peaks during Phase 2.

	*Barefoot*		*Foam*		*Shod*	
	Swing	Support	Swing	Support	Swing	Support
***ML force***	-0.005 ± 0.004^a^	0.072 ± 0.015^c^	-0.0002 ± 0.005^a^ ^,^ ^b^	0.070 ± 0.014^d^	-0.005 ± 0.003^b^	0.078 ± 0.015^c^ ^,^ ^d^
***AP force***	0.002 ± 0.006^e^	0.11 ± 0.02^g^	0.009 ± 0.006^e^ ^,^ ^f^	0.10 ± 0.02^g^ ^,^ ^h^	0.0008 ± 0.005^f^	0.11 ± 0.02^h^
***Vertical force***	0.07 ± 0.03	0.99 ± 0.03^j^ ^,^ ^k^	0.08 ± 0.03^i^	1.00 ± 0.03^j^ ^,^ ^l^	0.07 ± 0.02^i^	1.02 ± 0.04^k^ ^,^ ^l^

Values expressed as mean ± standard deviation. Repeated measures ANOVA pairwise comparisons:

^a^ p = 0.002,

^b^ p < 0.001,

^c^ p = 0.010,

^d^ p = 0.002,

^e^ p < 0.001,

^f^ p < 0.001,

^g^ p = 0.014,

^h^ p < 0.001,

^i^ p = 0.005,

^j^ p = 0.007,

^k^ p < 0.001,

^l^ p = 0.014.

**Table 4:** Force Peaks during Phase 3.

	*Barefoot*		*Foam*		*Shod*	
	Swing	Support	Swing	Support	Swing	Support
***ML force***		0.085 ± 0.012		0.085 ± 0.011		0.084 ± 0.013
***AP force***		0.15 ± 0.03		0.15 ± 0.02		0.15 ± 0.03
***Vertical force***		1.05 ± 0.04^a^		1.06 ± 0.04^a^ ^,^ ^b^		1.04 ± 0.04^b^

Values expressed as mean ± standard deviation. Repeated measures ANOVA pairwise comparisons:

^a^ p = 0.019,

^b^ p < 0.001.

In Phase 2 (Table 3), the ML swing-foot force peaks were significantly smaller in the foam condition than in the barefoot (p = 0.002) and shod conditions (p < 0.001). However, the ML support-foot force peaks were significantly larger in the shod condition than in the barefoot (p = 0.010) and foam (p = 0.002) conditions. In addition, the AP support-foot force peaks were significantly smaller in the foam condition than in the barefoot (p = 0.014) and shod (p < 0.001) conditions. In all directions, force peaks were larger under the support foot than under the swing foot, indicating a transfer of loading from the swing to the support foot.

In Phase 3 (Table 4), support-foot force peaks in the vertical direction were significantly larger in the foam condition than in the barefoot (p = 0.019) and shod conditions (p < 0.001).

**Fig 4:**
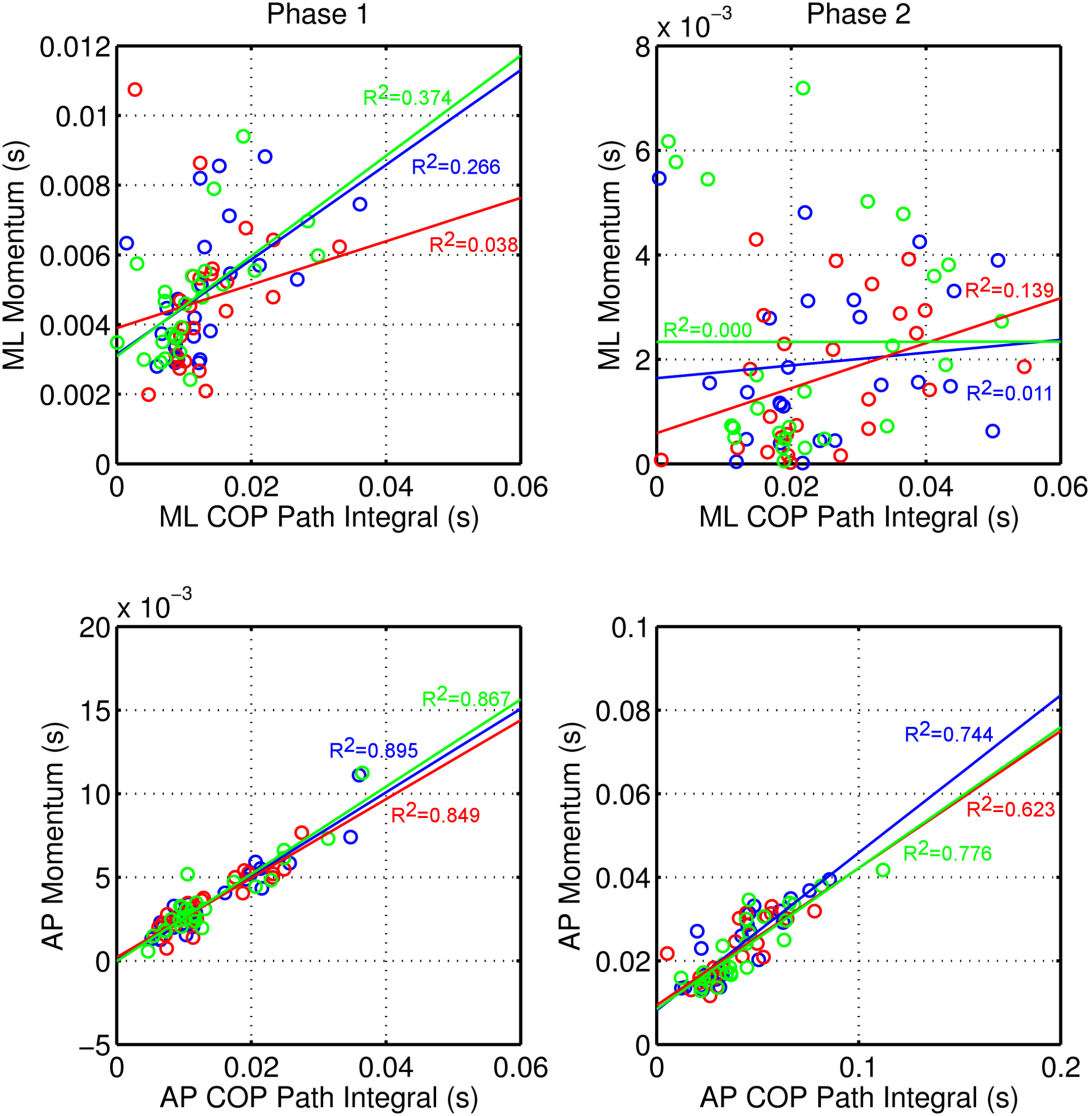
Force impulse x COP path integral graphs for Phase 1 and Phase 2 of gait initiation. Blue: barefoot condition; Red: foam condition; Green: shod condition. R2: R^2^ value for linear regression.

The comparisons for COP integral x force impulse among conditions are presented in Fig 4. Except when stated otherwise, all Spearman correlations were significant. In both the ML and AP directions, impulse varied with the COP integral, indicating that COP shift is responsible for impulse production. However, except for the AP direction in Phase 1, the intercepts are different from zero, indicating that force impulse is partially generated by other mechanisms and not exclusively by COP shift (Fig 4).

In Phase 1 COP, shift is responsible for only 3.8% (R^2^ = 0.038) of ML impulse production in the foam condition, whereas in the barefoot and shod conditions, COP shift is responsible for 26.6% (R^2^ = 0.266) and 37.4% (R^2^ = 0.374), respectively. In the AP direction, COP shift is responsible for 89.5% (R^2^ = 0.895), 84.9% (R^2^ = 0.849), and 86.7% (R^2^ = 0.867) of AP impulse production in the barefoot, foam, and shod conditions, respectively. The production of impulse in the AP direction is more dependent on COP shift than it is in the ML direction, and the influence of other mechanisms in the production of impulse are weaker (the intercepts are very near zero and R^2^s are larger).

In Phase 2, no significant correlations were found between the ML COP integral and force impulse (Spearman correlations: p = 0.214, p = 0.068, and p = 0.753 in the barefoot, foam, and shod conditions, respectively). The contributions of COP shift for impulse production are very low (R^2^ = 0.011, R^2^ = 0.139, and R^2^ = 0.000 in the barefoot, foam, and shod conditions, respectively). The ML impulse did not vary with the COP integral, and it was probably produced by mechanisms other than COP shift. In the AP direction, however, COP shift is responsible for 74.4% (R^2^ = 0.744), 62.3% (R^2^ = 0.623), and 77.6% (R^2^ = 0.776) of AP impulse production in the barefoot, foam, and shod conditions, respectively. Moreover, the intercepts are different from zero, suggesting that in the AP direction in Phase 2, other mechanisms are involved in the production of impulse in addition to those involved in the AP direction in Phase 1.

**Fig 5:**
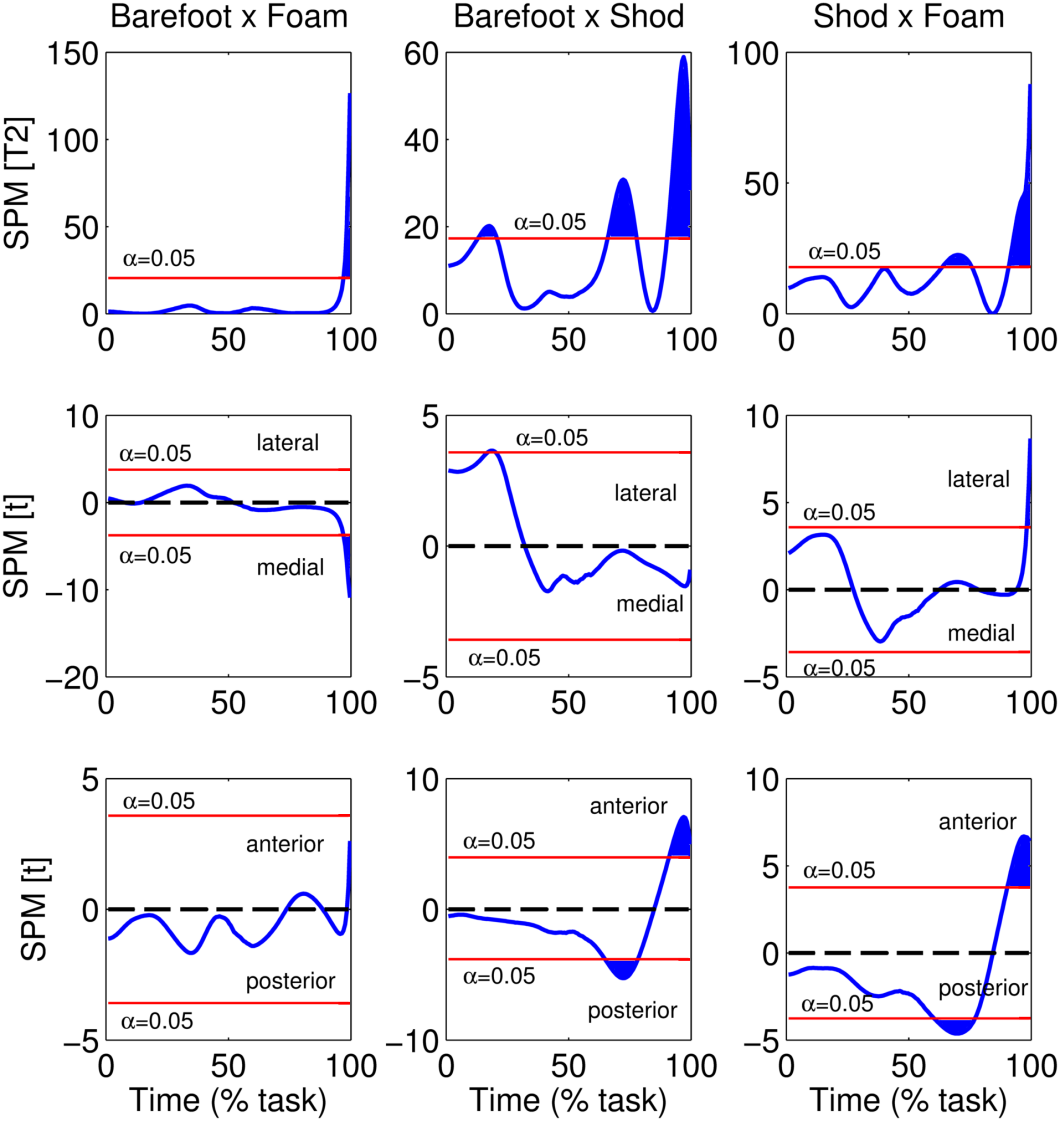
Hotelling’s *T* ^2^ trajectory of resultant COP (top graphs) and post hoc scalar *t* tests for COPx (middle graphs) and COPy (bottom graphs).

In the SPM analysis of the COP time series (Fig 5), differences were observed for all conditions, in which comparisons with shod condition exceeded the critical threshold for a greater proportion of the time series. These differences can be explained by the ML COP path in the last 10% (barefoot x foam) and at around 20% of the task (barefoot x shod), and by the AP COP path at around 65% and at the end of the task (in both barefoot x shod and shod x foam).

## Discussion

Comparing different surface conditions and the use of footwear, we expected to identify changes in gait initiation characteristics by analyzing COP descriptors, COP-force integrals, and COP time series. The main findings revealed that compared to the barefoot condition, both the foam and shod conditions significantly influenced gait initiation, but in different ways: the use of footwear affected gait initiation in both AP and ML direction, whereas foam affected the COP shift-impulse relationship in the ML direction during Phase 1.

Observing the resultant COP path variable, we do not consider the results in the AP direction observed in Phase 3 to be biomechanically or physiologically meaningful. The larger values found for the shod condition probably reflect the larger foot length resulting from the use of footwear.

The results in the ML direction observed in Phase 1 indicate that COP paths toward the swing foot and its corresponding velocities decreased in the shod condition, but not in the soft surface used in the study, when compared to the barefoot condition. Therefore, these results may imply that shoes limit intrinsic ankle/foot joint movements, modify the afferent input to the central nervous system, and consequently alter APAs particularly in the ML direction, whereas an unstable surface does not affect APAs.

However, no differences were observed in the AP direction for the Phase 1, probably because the deactivation of the plantar flexors and the activation of the anterior tibialis [31, 58] were not affected by either the shod or foam conditions and the backward COP shift was not altered. A similar conclusion can be drawn when analyzing the AP COP integral-force impulse relationship in Phase 1 (Fig 4, lower left graph). The initial backward COP movement increases the ground reaction force anteriorly, generating impulse in that direction. Because there is an increase in the ground reaction force through a COP shift rather than through a COM movement, the initial impulse necessary for taking a step is generated before the COM moves out of the support base, providing a more stable initiation of the gait. This pattern of ankle muscle activity and impulse generation is relatively constant [34] and is considered strong evidence of a central nervous system program of gait initiation [31, 58], and it does not appear to be altered in either the shod or foam conditions.

It is likely that the barefoot condition increases the degrees of freedom of ankle and foot motion and promotes higher plantar loading [60]. This mechanical feedback may enhance proprioception, which is desirable for postural adjustments and thus COP oscillations. The former reason may explain the greater COP path and higher velocity in the ML direction observed in the barefoot condition compared to the shod condition. This could mean that the somatosensory augmentation provided by the barefoot condition increased the perception of changes at the ankle and midfoot during gait initiation, providing more confidence to the subjects as they made ML adjustments and providing wider stability limits and more stable COP shift to the stance foot.

Conversely, as observed in the present study, the shod condition limited the joints’ range of motion, reduced the afferent input to the motor control system, and might have induced a restricted oscillation in the ML direction as a result. Studies have shown that compared to the barefoot condition, the use of footwear during gait limits the eversion/supination and the rate of eversion in late stance [61]. This limits the action of hip abductors, which are responsible for the lateral movement of the COP toward the swing limb [6] during Phase 1. The use of footwear constrained both the torsional and adduction range of foot motion. Shoes not only restrict the motion of the small foot joints but also appear to impose a specific foot-ankle motion pattern during push-off [61]. Furthermore, when compared to the barefoot condition, different kinds of footwear have been observed to alter the COP path under the foot [62]; this suggests that the use of shoes may have influenced ML COP oscillation in this study.

Supporting these conclusions, among all three conditions, the COP SPM analysis reveals that comparisons with shod condition exceeded the critical threshold for a greater proportion of the time series. During gait initiation Phase 1, differences can be observed in the ML COP component (Fig 5, middle graphs) between the shod and barefoot condition, supporting the conclusion that footwear alters the APA phase, as observed by resultant COP descriptor variables.

However, the SPM analysis also reveals other differences in the COP path for the shod condition—differences not revealed by resultant COP descriptor variables—particularly regarding around 65% of the task in the AP direction, corresponding to the swing foot’s toe-off and the beginning of the support foot’s heel rise. Furthermore, the differences observed in the anterior-posterior COP component in the last 10% of the task are probably not biomechanically meaningful, as discussed previously. However, despite the larger AP COP path in the shod condition during Phase 3, we observed a corresponding higher AP COP velocity, indicating that the subjects performed Phase 3 faster in the shod condition than in the barefoot and foam conditions.

COP descriptor variables and COP SPM analysis reveal significant differences in the foam condition compared to both the barefoot and shod conditions. Walking on two layers of 20-mm thick soft foam resulted in reduced velocity, cadence, step length, double-support time, and horizontal heel velocity at heel strike and in greater step width and toe clearance [63]. These alterations suggest adaptations to improve gait stability when walking on a soft surface. In the foam condition during Phase 3, the lower AP velocity and the corresponding AP COP path indicate that this phase has the longest duration, probably due to the greater toe clearance on the foam surface, as described by other gait studies [63]. The greater ML COP path in the foam condition in this phase can also be explained by greater toe clearance, which would lead to a greater oscillation toward the support foot and greater support-foot loading. All these COP changes in the foam condition may be due to an adaptation to stabilize the COP and guarantee postural stability instead of generating a great deal of forward impulse, similar to what has been observed in Parkinson’s patients [3], a population prone to locomotor problems.

The force peaks analysis reveals that the soft surface limited swing-foot loading during Phase 1 and support-foot loading during the Phase 2. The soft surface appears to alter the sensory information, which in turn limits the ability to properly grade the necessary adjustments during gait initiation [64]. However, greater vertical force peaks were produced in the foam condition during Phase 3, probably due to a sensation of the walkway sinking down during foot contact and to the fact that walking on a soft surface requires higher elevation of the foot during the swing phase [64], loading the support foot to a greater degree than in the barefoot and shod conditions. On the other hand, the greater force peaks in the shod condition during Phase 2 can be explained by the lower AP velocity resulting from the hardness of the plantar surface, which limits foot rollover, decreasing the contact time of the swing foot and leading to greater loading of the support foot.

The functional effectiveness of the gait initiation motor program with respect to impulse generation [34] can be analyzed by the linear regression between the COP integral and force impulse. In Phase 1, we observed that the impulse-generating capacity of the COP shift mechanism is diminished for the foam condition in the ML direction. The slope of the relationship between the time integral of the sideward COP shift and the amount of corresponding impulse generated is lower in the foam condition, indicating that less impulse is generated for each incremental change in COP displacement. Given that the size of the sideward COP shift was not different from that in the barefoot condition, this result is likely due to the COM moving sideward by a smaller amount in response to the initial force generated by the COP shift [34]. This decreased sideward COM movement in the foam condition during the initial stages of gait initiation is likely attributable to factors such as the perception of an unstable surface under the feet. Indeed, the confounding effects of the unstable foam surface may have limited the ability to scale the magnitude of preparatory postural adjustments for gait, as has been shown in children aged 4–6 [38].

Although the slope for the regression line for the ML COP shift-impulse relationship was lower in the foam condition, the intercept was higher. The regression-line intercept for the ML COP shift-impulse relationship represents the impulse that is generated in addition to that produced by the COP shift [34]. This result implies that in the foam condition, the subjects use other strategies in addition to the sideward COP shift to generate the sideward impulse needed to initiate gait.

In Phase 2, the ML force impulse generation is independent of COP shift, except in the foam condition, where the COP shift mechanism contributes to force impulse generation, although not significantly. As discussed previously, this result supports the idea that in the foam condition, the greater toe clearance required in the next phase leads to a greater oscillation toward the support foot and greater support-foot loading, with a greater ML COP path (Table 1).

In summary, these results indicate that the impulse-generating capacity of COP shift is limited when gait initiation is performed on a soft surface, probably due to sensory information indicating a nonrigid surface and to the amplitude of foot clearance required in this condition, so that the subjects use other strategies to load the body weight on the support foot. Our results confirm that foam condition imposes more challenging balance performance during gait initiation because it is believed that this surface decreases the reliability of somatosensory information from cutaneous mechanoreceptors of the plantar sole. The peculiar characteristics of the compliant surface in foam condition may have changed the impulse-generating strategy, especially in ML direction, responses well described for standing postural control [65].

It is possible that strategies other than the COP shift mechanism can be used to generate the impulse needed for gait initiation. Polcyn et al. [34] suggested that upon departing from a quiet-standing position, the resultant ground reaction force is displaced from its vertical position by shifting the upper body forward and toward the support foot. This strategy, however, would lead to an unstable body configuration, because it would result in the trunk being moved beyond the base of support. The COP shift mechanism is a more stable strategy, because it generates the initial impulse needed for gait initiation without requiring the upper body to be moved beyond the base of support, allowing subjects to maintain balance during the initial transition phase [34].

This study has some limitations that should be noted. We were not able to evaluate the movement of the foot inside the footwear, and the rigidity of the shoe was not controlled (each subject performed the task using his or her habitual athletic shoe). Because the data were collected using only force platforms, we could not infer what additional mechanisms are involved in force impulse generation. Therefore, it would be of interest for further studies to collect kinematic and EMG data to analyze the influence of shoe midsole hardness during gait initiation and the use of footwear in different populations, such as elderly people.

## Conclusions

In short, confirming our hypothesis, the use of footwear changes COP and ground reaction forces behavior especially in Phase 1 (the APA phase), and soft and unstable surfaces decrease the COP integral-impulse relationship. In the Phase 2, because of the greater instabilities on a soft surface, the foam condition results in more restricted responses, whereas in the Phase 3, the use of footwear benefits COP behavior in the AP direction.

Furthermore, SPM analysis of COP time series also reveals significant differences for the shod condition during the Phase 1 and the Phase 2.

Therefore, because the use of shoes changes the motor response of the subjects in the present study, this finding should be considered in balance and gait initiation studies in which the focus is not on the effect of footwear.

## Supporting Information

S1 Dataset Collected Data. (ZIP)
